# Prognostic value of PLA2R autoimmunity detected by measurement of anti-PLA2R antibodies combined with detection of PLA2R antigen in membranous nephropathy: A single-centre study over 14 years

**DOI:** 10.1371/journal.pone.0173201

**Published:** 2017-03-03

**Authors:** Franck Pourcine, Karine Dahan, Fabrice Mihout, Marine Cachanado, Isabelle Brocheriou, Hanna Debiec, Pierre Ronco

**Affiliations:** 1 Assistance Publique-Hôpitaux de Paris, Hôpital Henri Mondor, Department of Nephrology and Transplantation, Créteil, France; 2 Assistance Publique-Hôpitaux de Paris, Hôpital Tenon, Department of Nephrology and Dialysis, Paris, France; 3 Assistance Publique-Hôpitaux de Paris, Hôpital Saint Antoine, Department of Clinical Pharmacology and Unité de Recherche Clinique, Paris, France; 4 Assistance Publique-Hôpitaux de Paris, Hôpital Tenon, Department of Pathology, Paris, France; 5 Sorbonne Universités, Université Pierre et Marie Curie Univ Paris, Paris, France; 6 Institut National de la Santé Et la Recheche Médicale, Unit, Paris, France; University of Washington, UNITED STATES

## Abstract

**Introduction:**

Clinical course of membranous nephropathy (MN) is difficult to predict. Measurement of circulating anti-PLA2R autoantibodies (PLA2R-Ab) and detection in immune deposits of PLA2R antigen (PLA2R-Ag) are major advances in disease understanding. We evaluated the clinical significance of these biomarkers.

**Methods:**

In this 14-year retrospective study, we collected data from 108 MN patients and assessed the relationship between clinical course, PLA2R-Ab and PLA2R-Ag. We also assessed THSD7A status.

**Results:**

Eighty-five patients suffered from primary MN (PMN) and 23 patients from a secondary form. The median follow-up was 30.4 months [interquartile range, 17.7;56.7]. Among the 77 patients with PMN and available serum and/or biopsy, 69 (89.6%) had PLA2R-related disease as shown by anti-PLA2R-Ab and/or PLA2R-Ag, while 8 patients (8/77, 10.4%) were negative for both. There was no significant difference between these two groups in age at diagnosis and outcome assessed by proteinuria, serum albumin level and eGFR. Two of the 8 negative patients were positive for THSD7A. In patients with PLA2R related PMN, younger age, lower proteinuria, higher eGFR, and lower PLA2R-Ab level at baseline and after 6 months were associated with remission of proteinuria. Initial PLA2R-Ab titer ≤ 97.6 RU/mL and complete depletion of PLA2R-Ab within 6-months were significantly associated with spontaneous remission at the end of follow-up. In rituximab treated patients, lower PLA2R-Ab titer at initiation of treatment, and absence of PLA2R-Ab and higher serum albumin level at 3 months were significantly associated with remission. Noticeably, 81.8% of the patients who achieved remission completely cleared PLA2R-Ab. Depletion of PLA2R-Ab and increase of serum albumin level preceded the decrease of proteinuria.

**Conclusion:**

Assessment of PLA2R autoimmunity is essential for patient management. Combination of PLA2R-Ab and PLA2R-Ag increases diagnosis sensitivity. PLA2R-Ab titer is a biomarker of disease severity at initial assessment, and the kinetics of the antibody are significantly correlated to disease evolution.

## Introduction

Membranous nephropathy (MN) is one of the leading causes of nephrotic syndrome in adult. Some cases are associated with malignancy, infections, autoimmune systemic diseases, or drugs, but most are of a primary nature, being referred to as primary (PMN) and caused by autoimmunity against podocyte antigens, mainly the phospholipase A2 receptor (PLA2R) [1, 2].

The course of MN is mostly unpredictable. Although spontaneous remission occurs in one third of patients, another third will have a progressive loss of renal function, evolving to ESRD after 8 years, in the absence of specific treatment [3, 4, 5]. Treatment is still challenging and controversial because of potential toxicity and lack of a reliable prognosis marker. In the past, several studies have shown that immunosuppressive therapies as steroids and alkylating agents or cyclosporine could lead to remission of proteinuria and preservation of renal function [6–8]. However, immunosuppressive therapies are not innocuous, causing adverse events such as infections and malignancy [9]. Current challenges include identifying patients with a severe prognosis, treating them adequately, and evaluating efficacy of treatment as early as possible to adapt therapy to each patient and thus avoid unnecessary side-effects and costs. Treatment decision has long been based on a prospective follow-up of proteinuria, serum albumin level and renal function although these biomarkers are highly imperfect because they only indirectly reflect auto-immune activity [10]. Because clinical outcome in PMN patients is variable from remission to ESRD, reliable markers of immunological activity are of major interest to avoid risks of over- or under-treatment.

In 2009, the M-type phospholipase A2 receptor (PLA2R), a podocyte membrane glycoprotein, was identified as the first autoantigen involved in PMN in the adult [2]. Circulating PLA2R-Ab is detected in 70 to 80% of patients with PMN patients. Additional studies detected PLA2R-Ag in immune deposits in kidney biopsy [11]. Many studies reported that high titers of PLA2R-Ab are correlated with a lower risk of spontaneous or immunosuppressant-induced remission, a higher risk of nephrotic syndrome and of end-stage renal disease [12–18]. In a Chinese cohort, patients with undetectable PLA2R-Ab had a better prognosis than those with detectable PLA2R-Ab at onset, while persistence of PLA2R-Ag antigen in the deposits was associated with a higher risk of relapse [19].

More recently, another podocyte antigen, THSD7A, was identified accounting for less than 5% of PMN [20]. There is some indication that patients with anti-THSD7A antibody may have a higher risk of cancer [21].

We reviewed all patients referred to us over a 14-year period to revisit in a single centre the diagnostic, prognostic and predictive value of PLA2R-Ab, as well as the prevalence and potential cancer association of anti-THSD7A antibodies.

## Methods

### Patients and study design

We conducted a retrospective study of all patients with MN referred from January 2000 to January 2014 to the Nephrology department at the Tenon university hospital, Paris (France). Patients provided written informed consent for the use of their tissues and/or medical record data for research purposes (S1 Patient consent form). The study was approved by the IRB/Ethics Committee "CPP Ile de France IV", Hôpital Saint Louis, Paris (S1 CPP4). All renal pathology records were reviewed over a 14-year period to identify patients with histological diagnosis of MN. One hundred and eight patients were identified. Clinical data were obtained by checking patients’ medical records and included age, gender, blood pressure, date of renal biopsy, previous and concomitant supportive and immunosuppressant treatments (S1 Dataset and S1 Variables Listing). Biological data included urine protein to creatinine ratio (PCR), serum albumin, serum creatinine and estimated glomerular filtration rate (eGFR) by MDRD formula at the time of the initial renal biopsy and at each follow-up timepoint (diagnosis, 6 months or initiation of immunosuppressive treatment, 3 to 6 months after treatment, and end of follow-up). We defined the nephrotic syndrome as the association of proteinuria > 3.0g/24 h and a serum albumin < 30 g / L.

In 96 patients, we were able to retrieve sera sampled at the time of kidney biopsy, at around 6 months (median 6.3 months, extremes 4.75–9), at the initiation of immunosuppressive treatment if applicable, and at the end of follow-up.

At diagnosis, each patient was investigated with the aim to identify a cause for secondary MN such as malignancy, auto-immune disorder, infectious disease and a toxic cause. Primary membranous nephropathy was considered as a diagnosis of exclusion. Secondary membranous nephropathy was considered if any biological test or imaging procedure was positive at initial assessment or during follow-up. Initial evaluation included clinical examination, chest radiography, abdominal US for all patients and mammography if female. Blood samples were systematically tested for DNA antibodies and complement evaluation, HBV, HIV, HCV and syphilis serology. Patients were also asked to record their medications in search for a toxic cause. CT scan was performed in smokers and in patients over 50 years. Endoscopic investigation of colon and stomach was performed in patients over 50 years, or in case of familial history of colon cancer, or any manifestation suggestive of cancer.

All patients received supportive treatment including angiotensin-converting-enzyme inhibitor or angiotensin receptor blocker and lipid-lowering statin therapy.

**Fig 1:**
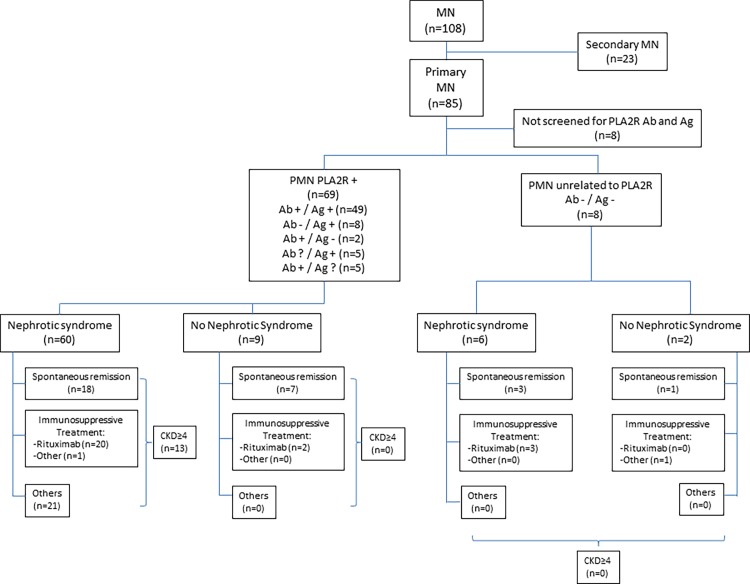
This flowchart depicts the patients who were enrolled in this retrospective study. They were divided into primary membranous nephropathy (MN) and secondary MN according to the criteria described in the Methods section. Seventy-seven of the 85 patients with primary MN could be screened for anti-PLA2R antibodies (Ab+ or Ab-) and PLA2R antigen in kidney biopsy (Ag+ or Ag-). Sixty-nine patients with PLA2R-related MN as defined by the presence of PLA2R-Ab and/or Ag, were then classified according to clinical presentation with or without the nephrotic syndrome, and further subdivided according to outcome and treatment. The 8 patients with primary MN not related to PLA2R immunity were classified in the same way. The number of patients reaching CKD stage 4 is also indicated.

The indication and choice of immunosuppressive specific treatment was left to the discretion of the physician in charge of the patient. Twenty-two patients were treated with rituximab (intravenous infusion of 375 mg/m^2^ body surface at D1 and D8 after intravenous premedication with 100 mg of methylprednisolone (Solumedrol®), 1 g of paracetamol and 5 mg dexchlorpheniramine (Polaramine®)). Two patients received alkylating agents plus glucocorticoids. The patients’ flow chart is shown in Fig 1.

Accordingly with 2012 KDIGO, complete and partial remission were defined as urinary protein excretion less than 30 mg/mM creatinine or less than 300 mg/mM creatinine (with at least 50% reduction versus baseline) respectively, accompanied by an increase or a return to normal of the serum albumin concentration and stable serum creatinine (≤ 25% variation). We considered both partial and complete remission. We also differentiated patients with an evolution to stage 4 or further CKD stage, defined as an eGFR< 30 mL/min with MDRD formula.

### PLA2R-Ab measurement

Circulating PLA2R-Ab was assessed on available serum samples by indirect ELISA. After sampling, all sera were immediately aliquoted, frozen and stored at –20°C. They were thawed only at the time of ELISA measurements. Previously unfrozen samples were never used for the tests. After thawing, all serum samples were tested for the presence of PLA2R-Ab total IgG antibodies using the ELISA test commercialized by EuroImmune AG (Lübeck, Germany). In brief, sera diluted to 1:100 were incubated with PLA2R already coated microplates and detected by incubation with anti-human IgG HRP conjugate. The final concentrations for each sample were calculated from the calibration curve extinction values plotted against the concentration for each calibrator. ELISA cut-off values were established according to manufacturers’ protocol and the results were considered as negative for < 14 RU/ml and positive for ≥ 14 RU/ml. The coefficients of variation (CV) were assessed by using 3 selected serum samples covering the measuring range. The intra-assay and inter-assay CVs were based on 20 measurements for each serum in one set or on threefold replica in ten sets, respectively. In our laboratory, the calculated intra- and inter-assay CVs are <4% and <9%, respectively. Up to five freeze/thaw cycles were found not to affect anti-PLA2R binding by ELISA.

### THSD7A-Ab measurement

THSD7A-Ab was assessed by an immunofluorescence test on HEK cells transfected with the full-length THSD7A cDNA, a kind gift of EuroImmune AG.

### Kidney biopsy study

All available paraffin-embedded kidney biopsies (n = 94) were analyzed by immunofluorescence using rabbit affinity-purified specific polyclonal anti-PLA2R antibodies (Atlas antibodies) followed by goat Alexa 488 conjugated anti-rabbit Fab IgG (Molecular Probes). THSD7A antigen was tested in PLA2R-negative biopsies using rabbit polyclonal antibodies (Atlas antibodies) followed by goat Alexa 488 conjugated anti-rabbit Fab IgG (Molecular Probes).

### Statistical analysis

Baseline characteristics of the study population were expressed as frequency and percentage for qualitative variables and as median (with interquartile range [IQR]) for continuous variables. Quantitative variables were compared by a Student’s t-test or a Wilcoxon rank-sum test, and categorical variables were compared by a Pearson's Chi-squaretest or a Fisher’s exact test. All PLA2R-Ab titers not achieving the 14 RU/ml detection threshold of the method were spiked at 0. PLA2R-Ab titer was considered as a continuous variable, as a binary variable (absence/presence),

Multivariate analysis using logistic regression were performed to identify potential predictive factors of remission. Proteinuria, serum albumin and PLA2R-Ab titer were log-transformed before being used as continuous variable. Following variables of interest were analysed in univariate analyses: age, sex, proteinuria, serum albumin, eGFR,PLA2R Ab titer at baseline and immunosuppressive treatment. A model was built using backward stepwise procedure with covariates selected in univariate analysis (P value<0.20).

All tests were two-sided and P values <0.05 were considered to indicate statistical significance.SAS^®^software (version 9.3) was used for statistical analyses.

## Results

### Study population

One hundred and eight patients with membranous nephropathy were identified. Clinical data are shown in Table 1 and Fig 1. Sixty-two percent of patients were male and median age at diagnosis was 55.2 years (IQR: 40.3; 67.7). Eighty-three patients (76.9%) patients presented with nephrotic syndrome, median PCR was 6.6 g /g (IQR 3.2; 10.8) and median serum albumin was 20.0 g/L (IQR 15.9; 26). Median eGFR was 78.8 mL/min (IQR 57.8; 99.0). Median follow-up was 30.4 months (IQR 17.7; 56.7). At the end of follow-up, 69 patients had achieved remission including 33 partial remissions and 36 complete remissions. Forty-seven patients experienced a spontaneous remission. Thirty-two patients still have a progressive disease.

**Table 1:** Characteristics of patients with primary and secondary MN.

	MN	PrimaryMN	Secondary MN	
Variable	N = 108	N = 85	N = 23	p-value
**Age at diagnosis(years)**	55.2 [40.3; 67.7]	54.0 [40.5; 65.1]	55.7 [35.6 ; 75.9]	0.3542
**Sex**				0.8965
Female	41 (38.0)	32 (37.6)	9 (39.1)	
Male	67 (62.0)	53 (62.4)	14 (60.9)	
**Proteinuria (g/d)**	6.6 [3.2; 10.8]	7.1 [3.5; 10.8]	4.7 [1.9 ; 12.0]	0.2724
**Serumalbumin (g/L)**	20.0 [15.9; 26.0]	20.0 [16.0; 26.0]	23.3 [11.9 ;30.0]	0.8690
**Nephrotic syndrome**				**0.0092**
Yes	83 (76.9)	70 (82.4)	13 (56.5)	
No	25 (23.1)	15 (17.6)	10 (43.5)	
**eGFR (mL/min)**	78.8 [57.8; 99.0]	74.0 [58.0; 99.0]	81.0 [51.2 ; 98.0]	0.9284
**PLA2RAg**				**<0.0001**
Positive	68 (73.9)	62 (83.8)	6 (33.3)	
Negative	24 (26.1)	12 (16.2)	12 (66.7)	
**PLA2R-Ab**				**<0.0001**
Positive	48 (55.2)	46 (67.6)	2 (10.5)	
Negative	39 (44.8)	22 (32.4)		
**Follow-up (months)**	30.4[17.7 ; 56.7]	31.8[18.0; 56.9]	22.0 [12.6 ; 49.2]	0.1029
**Remission**				
No remission	32 (317)	27 (33.8)	5 (23.8)	
Partial remission	33 (32.7)	28 (35.0)	5 (23.8)	
Complete remission	36(35.6)	25(31.3)	11 (52.4)	

Frequencies (percentage) or medians (interquartile range) are shown. Quantitative variables were compared by a Student’s t-test or a Wilcoxon rank-sum test, and categorical variables were compared by a Pearson's Chi-square test or Fisher’s exact test.

Eighty-five patients were diagnosed as PMN after an extensive work-up and 23 patients as secondary MN. At baseline, there was no difference in age (p = 0.35), proteinuria (p = 0.27), serum albumin (p = 0.87) and renal function (p = 1) between patients with PMN and secondary MN. During follow-up, all patients received supportive therapy consisting of angiotensin converting enzyme inhibitors or angiotensin receptor-blockers, irrespective of etiology.

### Characteristics of patients with secondary MN

Twenty-three patients presented with secondary MN, 9 had an auto-immune disorder (3 systemic lupus erythematous, 1 mixed connectivite tissue disease, 1 bullous pemphigoid, 1 Sjögren’s syndrome, 1 primary biliary cirrhosis and 2 sarcoidosis), 3 had an infectious disease (1 HBV, 1 syphilis, 1 mycobacterium avium), 10 had a malignancy, and 1 had a toxic cause (gold salt). Median age of patients with secondary MN was 55.7 years (IQR 35.6; 75.9). Among them, 60.9% were male (n = 14). Most patients (n = 13) had the nephrotic syndrome, median proteinuria was 4.7g/d (IQR 1.9; 12.0), and serum albumin level was 23.3 g/L (11.7; 30.9). Renal function at diagnosis was defined by a median eGFR of 81.0 mL/min (IQR 51.2; 98.0), (Table 1). Histological characteristics were similar to those of the patients with primary MN, except for the presence of a significant number of inflammatory cells infiltrating the glomeruli in a patient with a gastric carcinoma.

PLA2R serology was assessed in 16 patients. Two patients with MN associated with sarcoidosis had PLA2R antibodies. Glomerular PLA2R antigen deposits were detected in 6 patients (18 biopsies available), including 2 patients with a lung carcinoma, 1 patient with a Sjogren’s syndrome, 1 with chronic viral B hepatitis, and 2 with sarcoidosis. One patient with anti-THSD7A antibodies and THSD7A antigen in immune deposits had a gastric carcinoma.

The outcome of patients with secondary MN was as follows: 16 achieved partial or complete remission, 5 developed chronic renal failure, 2 reached end-stage renal disease. The 2 patients with anti-PLA2R antibodies and sarcoidosis achieved partial or complete remission after steroid treatment. Serological tests became negative before remission.

### Characteristics of patients with primary MN: comparison of clinical features between patients with PLA2R-related and PLA2R-unrelated MN

Eighty-five patients were diagnosed as having PMN. Characteristics of patients with PMN are shown in Table 1. Median age at diagnosis was 54.0 years (IQR 40.5; 65.1). Sixty-two percent of patients were male. Seventy (82.4%) patients presented with nephrotic syndrome at diagnosis. Median proteinuria was 7.1 g/d (3.5; 10.8) and median albumin level was 20.0 g/L (16.0; 26.0). Six patients (7.1%) had severe renal dysfunction, with an eGFR< 30mL/min at initial assessment.

Serological PLA2R-Ab status and PLA2R-Ag biopsy staining were available in 72 patients each. PLA2R-Ab was detected in 46 (64%) patients at diagnosis and in 56 (77.8%) patients during follow-up. Median ELISA titer of circulating PLA2R-Ab at diagnosis was 49.7 RU/mL (IQR; 0.0–287.7). PLA2R-Ag was revealed in immune deposits in 62 (83.8%) biopsies including 8 patients with negative PLA2R-Ab serology and 5 patients with no available serology during follow-up. Conversely, serology was positive in 2 patients without PLA2R-Ag on biopsy. Overall, 69 (89.6%) tested patients were categorized as having a PMN related to PLA2R autoimmunity, defined by at least one positive PLA2R-Ab detection in serum and/or positive PLA2R-Ag in immune deposits on biopsy evaluation. Among the patients without PLA2R related disease, two had THSD7A antibodies (2.7% % of available sera). When considering the whole time course of the disease, 79% (49/62) of the patients with PLA2R-Ag in immune deposits had at least one positive detection of PLA2R-Ab while 87.5% (49/56) of the patients with at least one positive detection of PLA2R-Ab had deposited PLA2R-Ag. Median follow up was 31.8 months (IQR 18.0; 56.9 months). At the end of follow-up, 66% of patients achieved remission and 34% still had disease activity.

We compared the outcome of patients with PMN related to PLA2R autoimmunity and patients without PLA2R autoimmunity. No statistical difference was observed between the two groups regarding baseline characteristics and outcome, except for a higher eGFR in PLA2R-negative patients at the end of follow-up (Table 2). Among the patients with PLA2R-related PMN, those without PLA2R antibodies at baseline (n = 11) achieved remission more often than patients with a positive serology (n = 43), the rates of remission being 90.9% (n = 10) and 58.1% (n = 25), respectively (p<0.0001).

**Table 2:** Comparison of patients with PLA2R-related and PLA2R-unrelated PMN.

	Positive anti-PLA2R (N = 69)		Negative anti-PLA2R (N = 8)			
	P50 [P25 ; P75]					
	n*		n*		p-value	
**Age at diagnosis (years)**	69	54.0 [41.4; 63.5]	8	50.0 [39.6; 67.0]	0.8810	^1^
**Follow-up (months)**	69	31.0 [19.0; 54.0]	8	40.1[25.8; 63.9]	0.4661	^1^
**At diagnosis**						
Proteinuria (g/d)	69	7.5 [4.0; 10.0]	8	8.7 [3.6; 14.5]	0.7144	^1^
eGFR (mL/min)	69	74.0 [57.0; 99.0]	8	87.4 [70.0; 105.5]	0.2529	^1^
Serum albumin (g/L)	69	20.0 [16.6; 26.0]	8	17.9 [16.4; 23.0]	0.5440	^1^
**End of follow-up**						
Proteinuria (g/d)	61	0.9 [0.3; 2.8]	8	0.7 [0.3; 1.7]	0.6710	^1^
eGFR (mL/min	62	70.5 [45.5; 88.0]	8	100.0 [73.0; 160.5]	**0.0374**	^1^
Serum albumin (g/L)	55	37.0 [32.0; 40.0]	8	36.0 [33.4; 40.0]	0.9067	^1^
Stage-4 CKD	62		8		0.3383	^2^
No	.	56(81.2)	.	8 (100)		^.^
Yes	.	13 (18.8)	.	0 (0)		^.^
Remission	64		8		0.2498	^2^
No	.	23 (35.9)	.	3 (37.5)		^.^
Yes	.	41 (64.1)	.	5 (62.5)		^.^

* number of patients.

Frequencies (percentage) or medians (interquartile range) are shown. Quantitative variables were compared by a Student’s t-test or a Wilcoxon rank-sum test, and categorical variables were compared by a Pearson's Chi-square test or Fisher’s exact test.

A subgroup of PLA2R-positive patients (n = 8) was negative for PLA2R-Ab in serum and positive for PLA2R-Ag in immune deposits. This subgroup was not different at diagnosis from PLA2R-Ab positive patients in term of age (56.2 years [48.7; 66.6] vs. 51.5years [42.2; 61.3], p = 0.5899), eGFR (86.5 ml/min [61.5;122.5] vs. 75.0 ml/min [57.5; 99.0], p = 0.5898) and serum albumin (22.8 g/L [18.2; 32.1] vs 20.1 g/L [16.5; 26.0], p = 0.3323), but proteinuria was lower (3.3 g/d [1.4; 4.6] vs 7.7 [4.2; 10.8], p = 0.0233. Spontaneous remission occurred in 6 (66.7%) patients in this subgroup as compared to 15 (35.7%) patients in the group of PLA2R-Ab positive patients, p = 0.0557.

### Comparison of clinical features between patients with PLA2R-related PMN achieving or not achieving remission

Of the 69 patients with PLA2R-related PMN, 62 had a follow-up >6 months and were considered for outcome analysis. Their median follow-up was 31.6 months (IQR 20.5; 55.4), (Table 3). At baseline, 41 (78.8%) patients with baseline available serum (n = 52) were PLA2R-Ab positive. Median PLA2R-Ab titer was 97.6 RU/ml (IQR; 23.8–344.6). At the end of follow-up period, 41 (66.1%) patients had achieved remission including 19 (30.6%) complete remissions and 22 (35.5%) partial remissions. Fifteen patients in the remission group had received an immunosuppressive therapy with anti-CD20 treatment, versus 7 patients in the group without remission.

**Table 3:** Outcome of patients with PLA2R-related PMN.

	Remission (N = 41)		No remission (N = 21)		
	n*		n*		p-value
**Age at diagnosis(years)**	41	51.4 [33.3; 59.3]	21	63.5 [53.9; 70.9]	**0.00017**
**At diagnosis**					
Proteinuria (g/d)	41	6.0 [3.1; 10.0]	21	8.5 [5.1; 13.1]	**0.0293**
eGFR (mL/min)	41	87.0 [64.0; 107.0]	21	61.0 [44.0; 84.0]	**0.0059**
Serum albumin (g/L)	41	19.6 [15.9; 26.0]	21	21.6 [16.8; 25.6]	0.6357
PLA2R-Ab (RU/mL)	35	44.4 [0.0 ; 188.9]	17	324.2 [157.5 ; 508.3]	**0.0018**
PLA2R Ab serology	35		17		0.0783
Positive		25 (71.4%)		16 (94.1%)	
Negative		10 (28.6%)		1 (5.9%)	
PLA2R-Ag	39		19		1
Positive		37 (94.9%)		19 (100%)	
Negative		2 (5.1%)		0 (0.0%)	
**At 6months**					
Proteinuria (g/d)	28	4.5 [2.4; 6.9]	15	8.0 [4.0; 13.3]	**0.0350**
eGFR (mL/min)	27	78.0 [56.0; 111.0]	15	33.0 [18.0; 52.0]	**<0.0001**
Serum albumin (g/L)	26	24.0 [20.0; 29.0]	15	22.4 [20.8; 30.0]	0.7568
PLA2R-Ab (RU/mL)	25	35.6 [0.0 ; 76.1]	8	291.2 [108.5; 606.3]	**0.0035**
Rituximab		15 (36.6)		7 (33.3)	P = 0.80
**End of follow-up**					
Proteinuria (g/d)	39	0.3 [0.3; 1.4]	18	6.0 [1.0; 8.0]	**< 0.0001**
eGFR (mL/min)	39	80.0 [66.0; 101.0]	19	28.0 [1.0; 48.0]	**< 0.0001**
Serumalbumin (g/L)	38	39.0 [35.0; 41.0]	13	35.0 [23.5; 36.0]	**0.0064**
PLA2R –Ab (RU/mL)	33	0.0 [0.0 ; 0.0]	13	49.3 [0.0 ; 207.7]	**0.0008**
PLA2R- Ab serology	33		13		**0.0017**
Positive		6 (18.2%)		9 (69.2%)	
Negative		27 (81.8)		4 (30.8%)	
Ab disappearance		20/ 23 (87.0)		3/12 (25.0%)	**0.0005**
CKD ≥ 4	40		20		**< 0.0001**
Yes		0 (0.0)		13 (61.9)	
No		41(100.0)		8(38.1)	

* number of patients.

Frequencies (percentage) or medians (interquartile range) are shown. Quantitative variables were compared by a Student’s t-test or a Wilcoxon rank-sum test, and categorical variables were compared by a Pearson's Chi-square test or Fisher’s exact test.

At diagnosis, patients reaching remission were younger (p = 0.0017), had lower proteinuria (0.0293), and better renal function (p = 0.0059) while serum albumin was not different between the 2 groups. Furthermore, a lower titer of PLA2R-ab at diagnosis and at 6-months follow-up was associated with remission status, respectively p = 0.0018 and p = 0.0008. PLA2R-Ab disappearance during follow-up was associated with remission, p = 0.0005.

By multivariate analysis, remission was associated with the decrease of proteinuria (adjusted OR 0.3; confidence interval 95%, [IC95%] 0.1–0.9; p = 0.0419) and with the decrease of PLA2R-Ab level (adjusted OR 0.4; IC95% 0.2–0.8; p = 0.0082).

### Comparison of clinical features between patients with PLA2R-related PMN achieving or not achieving spontaneous remission

Twenty-five (40.3%) PMN patients achieved spontaneous remission after a median follow-up of 34.3 months (IQR 23.7; 67.5) including 11 complete remissions (17.7%). Twenty-two (95.7%) patients showed PLA2R-Ag in immune deposits (23 available biopsies). Sixteen (66.7%) patients were PLA2R-Ab positive at baseline (Table 4) and median PLA2R-Ab value was 37.7 RU/mL (0.0–105.5) in the 24 patients achieving spontaneous remission at the end of follow-up with available serum. PLA2R-Ab titer (p = 0.0054) and rate of PLA2R-Ab positive patients (p = 0.0465) at initial assessment were lower in patients achieving spontaneous remission. During follow-up, 13 patients with spontaneous remission had an intermediary assessment of PLA2R-Ab after 6 months PLA2R-Ab was negative in 61.5% of the patients achieving spontaneous remission whereas only 9.1% of the patients with no remission were seronegative (p = 0.0017). PLA2R-Ab titer was lower in those patients who achieved spontaneous remission (0.0 RU/mL (0.0; 32.6) vs 74.1 RU/mL (37.4; 243.3), p = 0.0057).

**Table 4:** Comparison of the clinical features between patients with PLA2R-related PMN achieving or not achieving spontaneous remission.

	Spontaneous Remission (N = 25)		No spontaneous Remission (N = 37)		
	P50 [P25 ; P75]				
	n*		n*		p-value
**Age at diagnosis (years)**	25	51.5 [31.6; 59.8]	37	58.2 [43.4;68.8]	0.1164
**At diagnosis**					
PLA2R-Ag	23		35		1
Positive		22 (95.7)		34 (97.1)	
Negative		1 (4.3)		1 (2.9)	
PLA2R-Ab	24		28		**0.0465**
Positive		16 (66.7)		25 (89.3)	
Negative		8 (33.3)		3 (10.7)	
PLA2R-Ab (RU/mL)	24	37.7 [0.0; 105.5]	28	259.5 [42.2; 456.8]	**0.0054**
PLA2R-Ab ≤ 97.6RU/mL	24		28		**0.0054**
Yes		17 (70.8)		9 (32.1)	
No		7 (29.2)		19 (67.9)	
Proteinuria (g/d)	25	4.6[3.1; 7.5]	37	8.5 [5.0; 14.5]	**0.0051**
eGFR (mL/min)	25	81.0 [58.0; 107.0]	37	70.0 [53.0; 91.6]	0.37325
Serum albumin (g/L)	25	20.2 [15.9; 26.1]	37	19.5 [16.6; 24.4]	0.5024
**At 6 months**					
Proteinuria (g/d)	12	1.9 [1.0; 3.1]	31	7.2 [4.5; 12.1]	**0.0004**
eGFR (mL/min)	11	104.0 [56.0; 122.0]	31	55.0 [33.0; 77.0]	**0.0182**
Serum albumin (g/L)	10	29.0 [20.0; 32.0]	31	23.0 [20.0; 26.7]	0.1480
PLA2R-Ab (RU/mL)	13	0.0 [0.0; 32.6]	22	74.1[37.4; 243.3]	**0.0057**
PLA2R-Ab	13		22		**0.0017**
Positive		5 (38.5)		20 (90.9)	
Negative		8 (61.5)		2 (9.1)	
Rituximab		0.0 (0.0)		22 (59.5)	**<0.0001**
**End of follow-up**					
Proteinuria (g/d)	24	0.3 [0.3; 1.1]	33	1.4 [0.4; 6.0]	**0.0149**
eGFR (mL/min)	24	78.0 [65.0; 100.5]	34	63.5 [22.0; 82.0]	**0.0146**
Serumalbumin (g/L)	23	37.0 [33.0; 40.0]	28	37.0 [30.7; 40.5]	0.7414
PLA2R-Ab (RU/mL)	21	0.0 [0.0; 0.0]	25	0.0 [0.0; 106.8]	0.0503
PLA2R-Ab serology	21		25		0.0721
Positive		4 (19.0)		11 (44.0)	
Negative		17 (81.0)		14 (56.0)	
Ab disappearance	13	11 (84.6)	22	12 (54.5)	0.1390

* number of patients.

Frequencies (percentage) or medians (interquartile range) are shown. Quantitative variables were compared by a Student’s t-test or a Wilcoxon rank-sum test, and categorical variables were compared by a Pearson's Chi-square test or Fisher’s exact test.

Four patients experienced spontaneous remission despite persisting PLA2R-Ab at a median titer of 41.4 RU/mL (24.8–35.0) after a median follow-up of 45.0 months (17.0–67.0).

### Comparison of clinical features between PLA2R-related PMN patients treated with rituximab and achieving or not achieving remission

Twenty two patients with PLA2R-related MN were treated with rituximab which was infused at a median time of 8.0 months (IQR 7.0; 14.0) after diagnosis. Median follow-up after treatment was 17.0 months (IQR 7; 40). Proteinuria was7.6g/d (IQR 5.0; 12.6) at the onset of rituximab treatment and decreased to 0.5 g/d (IQR 0.3; 1.5) at the end of the follow-up (p<0.0001). During follow-up, renal function remained stable, and serum albumin increased from 18.0 g/l (IQR 15.0; 20.0) to 39.0 g/L (IQR 35.0; 41.0), p<0.0001. PLA2R-Ab titer decreased from 286.8 RU/mL (IQR 39.9; 405.3) to 0.0 RU/mL (IQR 0.0; 33.4) after treatment, (p = 0.0015).

At the end of follow-up, 15 patients (68.2%) had achieved remission, including 8 (36.4%) complete remissions and 7 (31.8%) partial remissions. We compared clinical features between patients reaching or not remission after rituximab. Achieving remission was associated with younger age (p = 0.0103), higher eGFR at diagnosis (p = 0.0222), and lower PLA2R-Ab titer (p = 0.0379), (Table 5). We also analyzed clinical features 3 months after the rituximab infusion. No difference was observed between the two groups regarding proteinuria rates or kidney function but the absence of PLA2R-Ab (p = 0.0222), the disappearance of PLA2R-Ab (p = 0.0120) and an early rise of serum albumin level (p = 0.0299) after rituximab treatment were associated with reaching remission.

**Table 5:** Predictors of outcome in patients treated with Rituximab.

	Rituximab Remission (N = 15)		Rituximab No Remission (N = 7)		
	P50 [P25 ; P75]				
	n*		n*		p-value
**Age atdiagnosis(years)**	15	46.7 [38.0; 58.4]	7	70.6 [62.1; 78.1]	**0.0103**
**At diagnosis**					
PLA2R-Ag	15		6		1.0
Positive		14 (93.3)		6(100)	
Negative		1 (6.7)		0 (0)	
Proteinuria (g/d)	15	7.6 [3.4; 15.2]	7	8.3 [5.1; 14.5]	0.6267
eGFR (mL/min)	15	91.6 [70.0; 139.0]	7	61.0 [38.0; 68.0]	**0.0222**
Serumalbumin (g/L)	15	18.2 [15.0; 20.0]	7	16.9 [12.0; 20.4]	0.5099
PLA2R-Ab (RU/mL)	11	44.4 [21.1; 286.8]	6	391.3 [365.0; 508.3]	**0.0379**
**At 6 months (Rituximab J0)**					
Proteinuria (g/d)	15	6.8 [4.5; 9.7]	7	12.6 [8.0; 14.0]	0.1055
eGFR (mL/min)	15	77.0 [56.0; 87.0]	7	33.0 [21.0; 52.0]	**0.0039**
Serumalbumin (g/L)	15	23.0 [19.0; 26.0]	7	20.8 [17.0; 21.1]	0.2565
PLA2R-Ab (RU/mL)	13	46.5 [29.5;85.0]	6	351.8 [145.0; 671.1]	**0.0322**
**3-months after treatment initiation**					
PLA2R-Ab	8		3		**0.0222**
Positive		0 (0.0)		2 (66.7)	
Negative		8 (100.0)		1 (33.3)	
PLA2-Ab disappearance	8		3		**0.0120**
Yes		8 (100.0)		0 (0)2 (100)	
No		0 (0.0)			
PLA2R-Ab (RU/mL)	8	0.0 [0.0 ; 0.0]	2	600.9 [134.9 ; 1066.8]	**0.0209**
Proteinuria (g/d)	15	2.9 [2.3 ; 4.3]	4	4.9 [3.0 ; 8.6]	0.3068
eGFR (mL/min)	15	83.0 [64.0 ; 98.0]	4	24.0 [19.0 ; 31.0]	**0.0085**
Serum albumin (g/L)	15	32.0 [25.7 ; 37.0]	4	18.0 [6.8 ; 24.0.]	**0.0299**
**End of follow-up**					
Proteinuria (g/d)	14	0.3[0.0; 1.5]	5	1.0 [0.5; 8.0]	0.1123
eGFR (mL/min)	14	82.0 [72.0; 102.0]	5	22.0 [9.0; 28.0]	**0.0050**
Serumalbumin (g/L)	14	39.5 [37.0; 41.0]	3	35.0 [6.7; 36.0]	0.0759
PLA2R-Ab (RU/mL)	11	0.0 [0.0; 0.0]	5	134.9 [47.4; 277.0]	**0.0112**
PLA2R-Ab	11		5		**0.0128**
Positive		1 (9.1)		4 (80.0)	
Negative		10 (90.9)		1 (20.0)	
CKD ≥ 4	15		7		**<0.0001**
Yes		0 (0)		6 (85.7)	
No		15(100)		1 (14.3)	

* number of patients.

Frequencies (percentage) or medians (interquartile range) are shown. Quantitative variables were compared by a Student’s t-test or a Wilcoxon rank-sum test, and categorical variables were compared by a Pearson's Chi-square test or Fisher’s exact test.

### Comparison of clinical features between patients with PLA2R-related PMN reaching or not reaching CKD stage ≥4

Thirteen (21.0%) patients with PLA2R-related PMN reached at least CKD stage 4 during follow-up (Table 6). Median follow-up for patients reaching or not reaching CKD stage *≥ 4* were 31.5 [23.5; 44.9] and 31.8 months [19.9; 57.0], respectively.

**Table 6:** Predictors of evolution to stage ≥4 CKD in patients with PLA2R-related PMN.

	CKD ≥ 4 (N = 13)		CKD < 4 (N = 49)		
	P50 [P25 ; P75]				
	n*		n*		p-value
**Age atdiagnosis(years)**	13	69.2 [58.2; 78.2]	49	51.5 [38.0;59.8]	**0.0024**
**Atdiagnosis**					
PLA2R-Ag	12		46		1
Positive		12 (100)		44 (95.7)	
Negative		0 (0)		2 (4.3)	
Proteinuria (g/d)	13	9.2 [7.7; 14.5]	49	6.0[3.5; 9.7]	**0.0300**
eGFR (mL/min)	13	61.0 [35.0; 68.0]	49	87.0 [58.0;101.0]	**0.0090**
Serum albumin (g/L)	13	19.5 [16.9; 26.0]	49	20.0 [16.0; 26.0]	0.9588
PLA2R-Ab (RU/mL)	11	377.3 [313.9; 754.6]	42	45.2[0.0; 210.4]	**0.0012**
PLA2R-Ab serology	11		41		**0.0930**
Negative		0 (0.0)		11 (26.2)	
Positive		11 (100)		30(73.2)	
**At 6 months**					
Protéinuria (g/d)	11	8.0[1.3; 13.3]	32	4.8[2.5; 7.4]	**0.1842**
eGFR (mL/min)	11	31.0 [11.0; 52.0]	31	77.0 [55.0; 109.0]	**0.0004**
Serum albumin (g/L)	10	22.0[19.0; 30.1]	30	24.0 [20.0; 29.0]	0.6713
PLA2R-Ab (RU/mL)	6	343.2 [72.0; 671.1]	29	35.0 [0.0; 82.8]	**0.0091**
PLA2R-Ab serology	6		29		0.1519
Negative		0 (0.0)		10 (34.5)	
Positive		6 (100)		19(65.5)	
**End of follow-up**		31.5 [23.5 ; 44.9]		31.8 [19.9 ; 570]	0.8162
Proteinuria (g/d)	11	1.0 [0.8; 6.4]	46	0.5[0.3; 1.8]	0.0578
eGFR (mL/min)	12	11.0 [1.0; 25.0]	46	79.0 [64.0; 100.0]	**<0.0001**
Serumalbumin (g/L)	7	35.0 [28.4; 38.0]	44	38.0 [33.0; 405]	0.3284
PLA2R-Ab (RU/mL)	9	49.3 [16.1; 277.0]	37	0.0 [0.0; 0.0]	0.0015
PLA2R-Ab serology	9		37		**0.0029**
Positive		7 (77.8)		8 (21.6)	
Negative		2 (22.2)		29 (78.4)	
Ab disappearance	9		26		**0.0030**
No		7 (77.8)		5(19.2)	
Yes		2 (22.2)		21 (80.8)	

* number of patients.

Frequencies (percentage) or medians (interquartile range) are shown. Quantitative variables were compared by a Student’s t-test or a Wilcoxon rank-sum test, and categorical variables were compared by a Pearson's Chi-squaretest or Fisher’s exact test.

At baseline, a higher level of proteinuria (p = 0.0300) and a higher titer of PLA2R-Ab (p = 0.0012) were significantly associated with reaching severe CKD. Similarly, we found a significant effect of PLA2R-Ab titer after 6 months on renal function at last follow-up (p = 0.0015). Moreover, disappearance of PLA2R-Ab during follow-up was associated with a better kidney function (p = 0.0030).

### Association of PLA2R-Ab titer with disease progression

Considering patients with PLA2R related PMN, statistical analyses showed a significant effect of initial positive serology and PLA2R-Ab titer on disease progression (lack of spontaneous remission, lack of induced remission and more advanced CKD). Among our population, 52 patients had a PLA2R serology available at diagnosis. In order to determine an antibody titer threshold for predicting patient outcome, we separated this population according to the median antibody titer at diagnosis. We then compared disease progression between patients with antibody titer > 97.6 RU/ml (n = 26) and those with PLA2R-Ab ≤ 97.6 RU/ml (n = 26), (Table 7). Patients with a higher PLA2R-Ab titer had a higher rate of CKD (p = 0.0022), a lower rate of global remission (p = 0.0011) and a lower rate of spontaneous remission (p = 0.0054).

**Table 7:** Comparison of disease progression between patients with PLA2R-Ab ≤ 97.6 RU/ml and > 97.6 RU/ml.

	PLA2R-Ab ≤ 97.6 RU/ml (N = 26)		PLA2R-Ab > 97.6 RU/ml (N = 26)		
	n (%)				
	n*		n*		p-value
**CKD ≥ 4**	26		26		**0.0022**
No	.	25 (96.2)	.	16 (61.5)	.
Yes	.	1 (3.8)	.	10 (38.5)	.
**Remission**	26		26		**0.0011**
No		3(11.5)		14 (53.8)	
Yes		23(88.5)		12 (46.2)	
**Spontaneous remission**	26		26		**0.0054**
No	.	9(34.6)	.	19(73.1)	.
Yes	.	17 (65.4)	.	7(26.9)	.

* number of patients.

Frequencies (percentage) or medians (interquartile range) are shown. Quantitative variables were compared by a Student’s t-test or a Wilcoxon rank-sum test, and categorical variables were compared by a Pearson's Chi-squaretest or Fisher’s exact test.

## Discussion

This study presents a global picture of the prevalence of secondary and primary MN in relation with PLA2R autoimmunity, in a single center, over a period of 14 years. Although this study was retrospective since January 2000, we could assess PLA2R-Ab and PLA2R-Ag in most sera and biopsies, respectively. We could also retrieve enough follow-up archival sera to investigate the dynamics of PLA2R-Ab during spontaneous and treatment (rituximab) induced remissions.

Although PLA2R autoimmunity is considered to be rare in secondary membranous nehropathy, it was detected in 6/23 patients including 2 with sarcoidosis and 1 with hepatitis B. Such associations which have already been described [22–24] suggest that immunological perturbations associated with these two diseases might trigger PLA2R autoimmunity although a fortuitous coincidence cannot be excluded. Ten patients had a cancer: 2 with a lung carcinoma had PLA2R-Ag in immune deposits, none had circulating PLA2R-Ab; 1 with a gastric carcinoma had circulating anti-THSD7A-Ab and THSD7A-Ag in immune deposits. Although the numbers are low, our study is compatible with a preferred association of THSD7A autoimmunity with a cancer-related MN as recently reported by Tomas et al [20, 21]. Apart from exceptional cases where THSD7A-Ag was found in the tumor and dendritic cells in a metastatic lymph node [20], the causality link between MN and the cancer is often difficult to establish. To our knowledge, we report the first case of PLA2R autoimmunity associated with Sjögren's syndrome where production of PLA2R-Ab might result from polyclonal B-cell activation.

This study confirms our previous findings suggesting that detection of PLA2R-Ag in immune deposits could be more sensitive than that of PLA2R-Ab in the serum [11, 25]. Indeed, although initial serum samples were taken at the time of kidney biopsy before immunosuppressive treatment, sensitivity of detection of PLA2R autoimmunity rose from 77.8% using serology to 89.6% using a combination of PLA2R-Ab assessment in serum and PLA2R-Ag detection in kidney biopsy. Moreover, we found that the 8 PLA2R-Ab negative, PLA2R-Ag positive patients had lower proteinuria compared to PLA2R-Ab positive patients. The observation that 7 of 8 patients achieved spontaneous remission strongly suggests that those patients had entered immunological remission. In other words, these patients define a subset with a better prognosis and should not be treated with immunosuppressive agents as also recently shown by a Chinese group [19].

Although it is important to separate patients with MN related to PLA2R who require a specific immunological monitoring, from those with PLA2R-unrelated MN, we could not detect a difference in clinical characteristics, proteinuria, renal function, albumin level and remission rates between the 2 groups of patients. This probably reflects shared pathophysiology mechanisms involving other antigens than PLA2R, including THSD7A and most likely other podocyte antigens which are still to be identified. The pathogenic effect of THSD7A-Ab was recently demonstrated in mice injected with THSD7A-Ab containing human sera [21] while such demonstration is still pending for PLA2R-Ab in the absence of a murine model.

In patients with PLA2R-related PMN, we showed that an initial positive serology and a high initial PLA2R-Ab titer were correlated with a higher level of proteinuria and a worse outcome, including a lower chance of spontaneous or treatment-induced remission, and a higher rate of severe chronic kidney disease. These findings are consistent with previous reports [12–18, 26, 27], which did not include the detection of PLA2R-Ag and thus did not differentiate patients immunized against PLA2R but without detectable antibodies, from those with PLA2R-unrelated MN possibly immunized against THSD7A or other podocyte antigens. There is only one study from China [19] where both PLA2R-Ab and PLA2R-Ag were assessed, with similar results as in our cohort. Actually, there is no biomarker predicting outcome in patients with PLA2R-unrelated PMN although isolated cases may suggest a correlation of THSD7A-Ab kinetics with clinical outcome [20, 27].

In our cohort, we also found that the kinetics of PLA2R antibodies were essential to predict disease outcome. In particular, patients who cleared PLA2R-Ab from the serum within 6 months had a greater chance of clinical remission than those with persistent positive serology. This finding also is consistent with previous studies [13, 26, 27]. In addition, we determined a threshold corresponding to the median PLA2R-Ab titer (97.6 RU/ml) which helped to predict disease outcome. However, this threshold should be interpreted with caution because of the very small increased odds of developing CKD4 or above although it could be clinically relevant considering the short follow-up period compared to natural history of the disease.

In patients treated with rituximab, we found that patients with a higher titer of PLA2R-Ab at initiation of therapy had a lower chance of remission, and that PLA2R-Ab disappearance was correlated to remission. It is interesting to note that there was no difference in age, gender, baseline proteinuria, serum albumin, renal function and PLA2R-Ab titer between patients who achieved spontaneous remission and those who achieved remission with rituximab. In other words, we still lack a specific marker of response to rituximab. These findings obtained in a retrospective study nicely corroborate those of our recent, prospective GEMRITUX trial where the addition of rituximab to supportive, antiproteinuric therapy was compared to antiproteinuric therapy alone [28]. Furthermore, we observed that an earlier rise of serum albumin was observed before a decrease of proteinuria, as in the GEMRITUX trial [28]. Whatever the mechanisms involving a systemic effect of rituximab on liver synthesis or a decrease of protein reabsorption in the proximal tubule, this observation is of great importance because dynamics of albumin levels may be early predictor of clinical response, particularly in those patients with PLA2R-unrelated MN without PLA2R-Ab in serum.

This study has several limitations intrinsic to retrospective studies. However, it provides important data on the impact of combined assessment of PLA2r-Ab and PLA2R-Ag on patients’ care. PLA2R-Ab has become an invaluable prognostic biomarker in patients with MN both in retrospective studies like this one, and in prospective studies like GEMRITUX. It may help define a subpopulation of patients who need early immunosuppressive treatment. The next step will be integration of this biomarker in KDIGO guidelines.

## Supporting information

S1 Patient consent form This document depicts the consent that was signed by each patient included in this study. (PDF)

S1 CPP4 This document depicts the Institutional Review Board Authorization. (PDF)

S1 Dataset This file depicts the data obtained in the cohort of patients with membranous nephropathy. Headings are depicted in S1 Variable listing.
(XLS)

S1 Variables Listing This file depicts the variables listed as headings in S1 Dataset. (XLSX)
