# Supplementary material for: Prognostic value of PLA2R autoimmunity detected by measurement of anti-PLA2R antibodies combined with detection of PLA2R antigen in membranous nephropathy: A single-centre study over 14 years
Source: PLoS One. 2017 Mar 3;12(3):e0173201. doi: 10.1371/journal.pone.0173201 (PMC5336294; doi:10.1371/journal.pone.0173201)
Supplement: S1 Patient consent form — (PDF) [file pone.0173201.s001.pdf]

Formulaire de consentement

de prélèvements réalisés au moment d'une biopsie rénale

Cadre réservé au service

Nom/prénom/identifiant du patient :

Etiquette patient	*Cachet du responsable du service*

Madame, Monsieur,

Dans votre cas, des indices d'une affection rénale ont été constatés. Afin d'obtenir plus d'informations au sujet de vos reins, un prélèvement de tissu rénal sera réalisé au cours d'une biopsie de rein. Les médecins qui vous traitent vous ont déjà donné des explications et des informations quant aux bénéfices potentiels, aux effets secondaires éventuels ainsi qu'à la réalisation du prélèvement de tissu rénal.

Cette biopsie sera réalisée lors de votre hospitalisation dans le Service de Néphrologie qui vous suit et des prélèvements sanguins et d'urines seront effectués simultanément.

Dans ce contexte, nous avons l'habitude de conserver systématiquement quelques millilitres de sérum, de plasma et d'urines ainsi que des cellules contenant de l'ADN. Cela représente au maximum 25 ml de sang supplémentaire prélevés. Nous conservons également un minime fragment du tissu rénal qui n'est pas utilisé pour le diagnostic de votre maladie rénale.

Ces prélèvements pourront éventuellement être utilisés dans le futur pour préciser le diagnostic de votre maladie rénale et/ou adapter votre traitement.

Une partie de ces prélèvements pourra, sauf opposition de votre part, être utilisée pour la recherche médicale, dans le respect de la confidentialité.

Grâce à leur utilisation, nous espérons mieux comprendre dans le futur les mécanismes responsables des maladies rénales. Ces études sont réalisées dans le cadre de différents programmes de recherche nationaux ou internationaux.

PARAPHES DU PATIENT PARAPHES DU MEDECIN

Les prélèvements de sang seront envoyés à la tumorothèque du service d'Anatomo-pathologie de l'Hôpital TENON (Mr Emmanuel Roux, responsable de la tumorothèque de TENON, Service d'Anatomopathologie, bâtiment Achard, Hôpital TENON, 4 rue de la chine, 75020 PARIS).

Les biopsies rénales seront acheminées et stockées dans l'Unité INSERM UMRS_702 sous la responsabilité de Mme Chantal Jouanneau (responsable de la tissuthèque rénale, UMR_S702, Bâtiment de recherche, Hôpital Tenon, 4 rue de la Chine, 75020 PARIS)

Les analyses effectuées dans le cadre de nos études n'entraveront en rien les autres examens nécessaires à votre traitement.

Dans le cas où vous ne seriez pas d'accord, cela ne modifiera en rien la qualité de votre prise en charge et des soins qui vous sont prodigués. Vous pouvez également revenir sur votre consentement à tout moment sans que vous ayez à fournir des raisons et sans qu'il en résulte un quelconque préjudice pour vous.

Je soussigné(e), M(me)………………………………………………………………………… accepte que l'on conserve un prélèvement de tissu rénal, sanguin et d'urines, en vue d'éventuelles études ultérieures et je consens à ce que des examens portant sur mon patrimoine génétique soient réalisés dans le cadre des explorations biologiques nécessaires à l'étude de la pathologie pour laquelle je suis hospitalisé à l'hôpital Tenon.

Patient	***Médecin recueillant le consentement***
Fait à Paris, le / /<br>*Signature :*	*Nom du médecin : Dr ………………..*<br>*Fait à Paris, le / /*<br>*Signature :*

- Conformément à la loi (art. 16-1 et 16-6 du code civil, ce prélèvement ne pourra être cédé à titre commercial, ni donner lieu à une rémunération à votre bénéfice. Il pourra être utilisé pour des recherches effectuées en partenariat avec un ou plusieurs organismes publics ou privés.
- Les données médicales associées au prélèvement seront réunies sur un fichier informatique permettant leur traitement automatise dans le cadre des recherches. Vous disposez a leur égard d'un droit d'accès, de rectification et d'opposition conformément à la loi.
- Les informations portées sur ce document sont confidentielles et couvertes par le secret médical. A aucun moment, les données personnelles qui y figurent n'apparaîtront lors de la publication des résultats des travaux de recherche.
