# Supplementary material for: Prognostic value of PLA2R autoimmunity detected by measurement of anti-PLA2R antibodies combined with detection of PLA2R antigen in membranous nephropathy: A single-centre study over 14 years
Source: PLoS One. 2017 Mar 3;12(3):e0173201. doi: 10.1371/journal.pone.0173201 (PMC5336294; doi:10.1371/journal.pone.0173201)
Supplement: S1 CPP4 — (PDF) [file pone.0173201.s002.pdf]

Comité de Protection des Personnes Ile de France IV

Agrément du Ministère de la Santé et des Solidarités
arrêté du 12 juin 2006 (J.O. 27 juin 2006)

Hôpital Saint-Louis
Porte 5 du carré Historique
1 avenue Claude Vellefaux
75475 Paris Cedex 10

Responsable administrative :
Mme I. SCAGLIA
Tél. : 01.42.38.92.88
Fax : 01.42.38.92.98
cpp.iledefrance4@orange.fr

Président :
Dr J.-P. CESARINI
Vice-président :
M. Marc BORAND
Secrétaire générale :
Mme M. ASTRIE-BELLICK
Secrétaires générales adjointes :
Mme B. LEHMANN
Dr S. KLOUCHE
Trésorier :
Pr O. CHASSANY
Trésorière Adjointe :
Mme C. MASCRET

Membres du Comité :
Collège I
Médecins et chercheurs
O. CHASSANY
P. CHAUMET-RIFFAUD
J-P. CESARINI
E. CAROSELLA
S. KLOUCHE
M.-H. DIZIER
B. PAPP
F. ADNET
P. GOLDSCHMIDT
Pharmacien hospitalier
B. LEHMANN
Infirmière
C. DELETOILLE-LANDRE
M. ASTRIE-BELLICK

Collège II
Questions éthiques
J.-C. KRZYWKOWSKI
Psychologue
L. LACOSTE
Travailleur social
M. BORAND
Compétence juridique
C. MASCRET
P. A. DUMAS
P. AUBRY
M. BOUCHER
Associations de maladies et d'usagers
M. BERNARD-HARLAUT
M. TROUGOUBOFF
A.M MASURE

Institutional Review Board
Agrément de : US Department of Health and Human Services
(n° IRB 00003835)

Institutional Review Board
Agrément de : US Department of Health and Human Services (n° IRB 00003835)

Paris, le 14 mars 2013

Hôpital TENON
Pr P. RONCO
Service de nephrologie
4 rue de la Chine
759 70 Paris Cedex 20

Madame, Monsieur,

Le Comité a bien reçu votre courrier du 31 janvier 2013 concernant l'étude intitulée :

Protocole 2013/08NICB
« Etude sérologique et génétique chez les patients suivis pour une glomérulonéphrite extra membraneuse. »

Gestionnaire : SERVICE DE NEPHROLOGIE ET DIALYSES de l'HOPITAL TENON
Clinicien coordonnateur : Pr RONCO (Hôpital Tenon, Service de néphrologie et dialyses)

L'objectif principal est d'étudier la prévalence et l'évolution des anticorps anti-PLA2R chez les patients de la cohorte.

Le Comité a examiné les informations relatives à ce projet lors de la séance du 28 février 2013 et a émis une demande motivée d'informations complémentaires.
Membres présents : Dr J.-P. Cesarini (I), Pr O. Chassany (I), Dr E. Carosella (I), Mme C. Deletoille (I), Dr S. Klouche (I), Mme B. Lehmann (I), Mr B. Papp (I), Mme M. Bernard-Harlaut (II), Mr M. Borand (II), M. J.-C. Krzywkowski (II), Mme L. Lacoste (II), Mme C. Mascret (II), Mme A. M. Masure (II), Mme M. Astrie-Bellick (I), Mme M.H Dizier (I).

Suite à l'examen des documents soumis, le Comité confirme qu'il s'agit d'une étude non interventionnelle, n'entrant pas dans le champ d'application de la loi n°2004-806 du 9 août 2004 relative à la politique de santé publique.
Le CPP approuve les documents d'information.

Dr Jean-Pierre CESARINI
Président

- ☐ Votre courrier du 31/01/2013
- ☐ Protocole version 1 du 10/12/2012
- ☐ Résumé version 1 du 10/12/2012
- ☐ Liste des investigateurs version 1 du 10/12/2012
- ☐ Lettre d'information et consentement pour conservation des prélèvements avant biopsie rénale version pas datée
- ☐ Lettre d'information et consentement pour conservation des prélèvements réalisés dans le cadre du suivi de votre glomérulopathie extra membraneuse version pas datée
- ☐ Votre courriel de réponse du 14/03/2013
